# An Ensemble Approach to Predict the Pathogenicity of Synonymous Variants

**DOI:** 10.3390/genes11091102

**Published:** 2020-09-21

**Authors:** Satishkumar Ranganathan Ganakammal, Emil Alexov

**Affiliations:** 1 Department of Healthcare Genetics, Clemson University, Clemson, SC 29634, USA; ealexov@clemson.edu; 2 Department of Physics and Astronomy, Clemson University, Clemson, SC 29634, USA

**Keywords:** synonymous variants (sSNVs), random forest (RF), pathogenicity prediction, variant of unknown significance (VUS)

## Abstract

Single-nucleotide variants (SNVs) are a major form of genetic variation in the human genome that contribute to various disorders. There are two types of SNVs, namely non-synonymous (missense) variants (nsSNVs) and synonymous variants (sSNVs), predominantly involved in RNA processing or gene regulation. sSNVs, unlike missense or nsSNVs, do not alter the amino acid sequences, thereby making challenging candidates for downstream functional studies. Numerous computational methods have been developed to evaluate the clinical impact of nsSNVs, but very few methods are available for understanding the effects of sSNVs. For this analysis, we have downloaded sSNVs from the ClinVar database with various features such as conservation, DNA-RNA, and splicing properties. We performed feature selection and implemented an ensemble random forest (RF) classification algorithm to build a classifier to predict the pathogenicity of the sSNVs. We demonstrate that the ensemble predictor with selected features (20 features) enhances the classification of sSNVs into two categories, pathogenic and benign, with high accuracy (87%), precision (79%), and recall (91%). Furthermore, we used this prediction model to reclassify sSNVs with unknown clinical significance. Finally, the method is very robust and can be used to predict the effect of other unknown sSNVs.

## 1. Introduction

The advances in the field of genomics have offered a wide range of opportunities and challenges to investigate the role of genetic variants in diseases. The single-nucleotide variants (SNVs) play a major role in altering various biological processes such as transcription, translation, and signal regulation [1]. The two major types of SNVs are missense or non-synonymous variants (nsSNVs) and synonymous variants (sSNVs). The nsSNVs alter amino acid residues that may lead to disruption of protein function, whereas the sSNVs do not alter the corresponding amino acid residue and thus have no direct effect on the protein and its function [2,3,4,5,6].

The impact caused by nsSNVs on resulting proteins make them preferred candidates for investigation in diseases compared to sSNVs. sSNVs have been identified to impact protein conformation that in turn affects the post-translational processes such as splicing and RNA folding, thus contributing substantially to phenotypic traits [7]. The sSNVs are referred to as “silent mutations”, but from literature, it has been observed to be associated with almost 50 different diseases/phenotypes [8].

Many computational methods have been developed for the characterization of missense (or nsSNVs) variants to either predict disease causing ability, such as Sorting Intolerant From Tolerant (SIFT) [9], Protein Variation Effect Analyzer (PROVEAN) [10], Rare Exome Variant Ensemble Learner (REVEL) [11], Combined Annotation Dependent Depletion (CADD) [12], Eigen [13], etc. or to predict their biophysical or biochemical effects [2,3,14,15,16]. At the same time, only a handful of computational methods are available to predict the pathogenicity of sSNVs, such as SilVA (Silent Variant Analyzer) [17], DDIG-SN (Detecting Disease-causing Genetic Synonymous variants) [18], TraP (Transcript-inferred Pathogenicity) [19], and IDSV (Identification of Deleterious Synonymous Variants) [20]. All these tools have been constructed using concepts of classification algorithms such as random forest (RF) and support vector machine (SVM), and also by utilizing various features encompassing splicing, conservation, and sequence (RNA and DNA) properties (Table 1) of the sSNVs extracted from various data sources, such as ClinVar [21], 1000 Genomes [22], HGMD [23] (Stenson et al., 2014), dbDSM [24], and VariSNP [25].

ClinVar is a prominent database of SNVs and indels that have been detected via various molecular genomics methods (such as Sanger, whole genome sequencing (WGS), or whole exome sequencing (WES)) annotated with clinical relevance based on the guideline by the American College of Medical Genetics and Genomics (ACMG), such as pathogenic (a variant that significantly contributes to the development of disease) or benign (a variant that does not cause disease), likely pathogenic/benign (a variant that has a high likelihood of being categorized as pathogenic or benign), or a variant of uncertain significance (VUS) (a variant with insufficient information to be classified as a benign or a pathogenic) [26]. The characterization of an sSNV’s clinical relevance either as pathogenic (disease-causing) or benign (non-disease-causing or benign) poses many challenges. The main hurdle is that sSNVs do not involve protein sequence changes; therefore, a direct assessment of the effect on protein function is not applicable. Because of that, the existing computational approaches assess pathogenicity using features such as conservation score, sequence, and splicing properties of the corresponding exome/intronic DNA sequence or RNA.

In this study, we have implemented the concept of an ensemble predictor by incorporating sSNV features (such as splicing, conservation, and sequence properties) along with scores from other in silico predictors (non-missense specific) such as CADD and Eigen. The ensemble predictor is built using a random forest (RF) classification algorithm with the top 20 ranked features, and it is shown to outperform existing approaches to distinguishing pathogenic from benign variants. The developed ensemble classification algorithm is applied to reclassify sSNVs categorized as variants of unknown significance (VUS) and conflicting interpretations.

## 2. Materials and Methods 

### 2.1. Dataset

The ClinVar database was our primary data source, and the repository was downloaded in variant call file (VCF) format from the FTP site (https://www.ncbi.nlm.nih.gov/clinvar/) [27]. Within the repository, 243 sSNVs are annotated as pathogenic and 9109 sSNVs are annotated as benign, and at the same time have allele frequency (AF) < 0.05 in the 1000 Genomes population database. Since there are more annotated benign sSNVs than those annotated as pathogenic sSNVs, and we wanted to have a balanced dataset, we randomly selected 243 benign sSNVs five times from the pool of 9109 benign sSNVs, and thus created five datasets, each having the same 243 pathogenic sSNVs and a different set of benign sSNVs (Supplementary File 1).

For testing and benchmarking our method, the datasets above were split into 90% and 10% (where 90% was a training dataset and 10% was a test dataset) (Supplementary File 2). We also constructed an independent dataset that encompassed all the ClinVar sSNVs classified as variants of unknown significance (VUS) or conflicting interpretations. 

We also used another test dataset, called Sauna et al. dataset, which was obtained from their curated literature [8] and consists of 23 sSNVs (GRch37 V) in 17 different genes that are associated with 16 different disorders (such as asthma, cancer, schizophrenia, etc.) (Supplementary File 3).

### 2.2. Feature Extraction

Our ensemble classification method was built on the same idea as the meta in silico predictor that combines both independent (or standalone) features along with a few already existing in silico prediction algorithms. The method uses 29 features corresponding to five major classes: in silico prediction score, conservation, codon usage biases, splicing, and sequence properties. Below we describe these classes in detail.

In silico prediction score: The in silico class consists of three major tools—CADD, Eigen, and Transcript-inferred Pathogenicity (TraP). CADD and Eigen were identified as the two top-performing in silico algorithms in previous SNV pathogenicity characterizations compared to conventional methods such as Polyphene, SiFT, and others [28]. CADD evaluates the deleterious nature of the SNV using various genomic features such as gene and sequence content, epigenetic measurements, and functional predictions [12]. Eigen uses an unsupervised method that evaluates the pathogenicity of an SNV based on the estimates of divergent functional scores [13]. TraP (V3) evaluates the pathogenicity of an SNV by determining the ability of the SNV to damage the final transcript [19].

The conservation feature was obtained from three main sources: GERP++ (Genomic Evolutionary Rate Profiling) [29], PhyloP (phylogenetic *p*-values) [30], and PHAST (Phylogenetic Analysis with Space/Time models) 100-way vertebrates conservation [31]. The GERP++ score was obtained as part of the SilVA preprocessing step, whereas the PhyloP and PHAST data were downloaded from the UCSC genome browser [32].

The quantified codon usage biases group includes RSCU (relative synonymous codon usage) and dRSCU (estimated change in RSCU caused by a mutation) that are calculated based on frequencies of observed codon across species obtained from the codon usage database annotated by the SilVA preprocessing step. 

The sequence property features include the presence or absence of mutation at the CpG site. The CpG_exon provides the ratio of observed and expected CpG content of the exon (due to mutation) along with the relative distance of the variant from pre- and mature-mRNA (f_premrna, f_mrna) available from the SilVA preprocessing step.

The splicing feature class consists of a total of 17 features, out of which 15 (such as MES, MES − KM, dMES, MES−, MES+, FAS6+, FAS6−, MEC−MC, MEC−CS, PESS−, PESS+, PESE−, PESE+, SR−, SR+) were also extracted as a part of the SilVA preprocessing step, and 2 features (dpsi, dpsiz) were extracted from the SPIDEX data resource Annovar repository [33]. Table 1 provides a detailed description of the features that we have extracted and used for this classification method.

### 2.3. Feature Selection and Ranking

After extracting all 29 features, we evaluated the best set of features that provides a high ability to differentiate the SNVs in our training dataset into pathogenic or benign, based on the statistics collected from the confusion matrix. For the feature selection process, we used the “ranker” option located under the classifier attribute evaluation method in Weka (v3.8.2) [34]. The ranker method is an explicit method that ranks the attributes based on relevance. The selection of the best set of features was done based on the AUC (area under the ROC curve) and accuracy in classifying the validation data.

### 2.4. Classification Model Selection and Evaluation

We built a machine learning supervised classification model using two prominent algorithms: random forest (RF) and Naive Bayes (NB). We applied them to our training set to compare their performance to differentiate between pathogenic and benign sSNVs. 

We used Weka [34] to build these two classification models and ran 10-fold cross-validation. The statistics obtained from the cross-validation, such as accuracy, precision, recall, F-measure MCC (Matthews correlation coefficient), and AUC (gives the ratio between true positive prediction rate to false positive prediction rate) (Table 2), were used to select the best model for classifying the test data. 

## 3. Results

### 3.1. Selection of Classification Algorithm

After collecting all of the 29 features, we used them to identify the best machine learning supervised classification method that can differentiate the pathogenic and benign variants. We evaluated two prominent algorithms, namely random forest (RF) and Naive Bayes (NB). We used Weka software and performed 10-fold cross-validation on the training datasets for both RF and NB (Table 3). One can see that RF outperformed NB by all measures, and this was true for all five test datasets. Because of that, in the rest of the paper we report results obtained with RF only. The next question to address is which model of RF is the best. It is expected that if there is no bias toward selecting the benign cases, the RF should generate similar results for all five training datasets (Table 3). The results were somehow similar but not identical, which we used to select the best model. Among the five training sets, Training Set 2 and Training Set 3 showed high accuracy and were selected for further investigation. In this further investigation, we wanted to select a set that performed the best while using fewer features, in order to reduce plausible overfitting. Thus, we subjected Sets 2 and 3 to the same 10-fold cross-validation procedure described above while using the top 10, 15, and 20 ranked features. Results are shown in Supplementary File 4. One can see that 20 features provided the best performance, and the model trained and tested with Set 2 outperformed the results obtained with Set 3 (the selection of features is further discussed in the next paragraph). Thus, Model 2 (the model trained on Set 2) was elected as the best model and used in the rest of the paper.

### 3.2. Feature Selection

**Figure 1:**
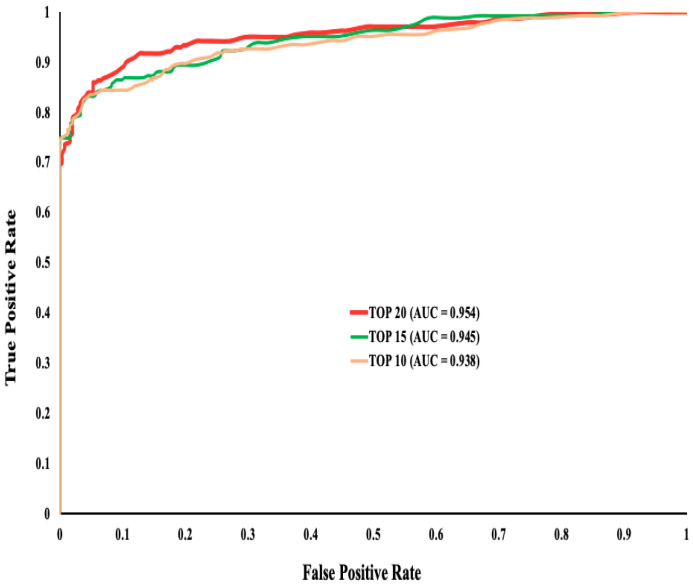
The ROC curve for evaluating the performance of the top (10, 15, 20) ranked features. Though the AUC is very close between all three sets, the top 20 features had better accuracy compared to the other two sets.

As mentioned above, we evaluated the performance of the top 10, top 15, and top 20 ranked features to seek the best predictive performance. We used 10-fold cross-validation to implement the RF classification method on the training dataset and obtained all the performance statistics for these three sets (Supplementary File 4). We observed that the top 20 ranked features outperformed all top 10 and top 15 feature sets with an accuracy of 86%, along with a higher AUC (Figure 1).

The selected top 20 features included 3 in-silico predictors (CADD, Eigen, and TraP(V3)), 2 conservation scores (GERP++, PhyloP), 10 splicing features (MES, dMES, MES+, MES−, MES-KM, dpsi, dpsiz, PESE+, SR+, SR−), 2 codon usage biases features (RSCU, dRSCU), and 3 sequence properties (CpG, CpG_exon, f_premrna) (Supplementary File 2). 

### 3.3. Benchmarking against other Methods Using Test Datasets

After identifying the best feature set (the top 20 features) and classification algorithm (RF) based on the training dataset, we evaluated the performance of our ensemble prediction method by splitting up the data into 90% training data and 10% test data and running 100 iterations. We also performed similar evaluations using the features and scores from other similar sSNV classification tools such as TraP(V3), SiLVA, and CADD.

**Figure 2:**
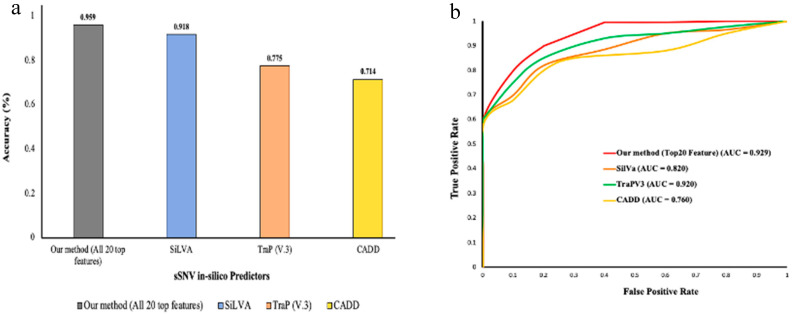
(**a**) Bar plot shows the distribution of accuracy obtained by each compared method on a known test dataset; (**b**) ROC curves for the same methods.

We observed that our method performed with 96% accuracy compared to the other sSNV pathogenicity prediction methods such as SiLVA, TraP(V3), and CADD with their accuracy ranging between 70% and 92% (Figure 2). 

### 3.4. Reclassification of Uncategorized sSNVs

In addition to testing and benchmarking our algorithm on ClinVar pathogenic and benign sSNVs, we performed a “blind” test on 23 synonymous variants obtained from previously published data [8]. Only 6 out of 23 sSNVs were classified as either benign or likely benign in ClinVar and were considered benign (TN). We observed that the six sSNVs previously annotated as likely benign/benign were also classified as benign by our algorithm. We were also able to identify two sSNVs (rs2069763 and rs1130569) with known associations to cervical/vulvar cancer and Alzheimer’s disease that were categorized as pathogenic variants (Supplementary File 3).

We also applied our algorithm to categorize the sSNVs in ClinVar that have been annotated as either VUS or conflicting interpretations. The results are shown in Supplementary File 5. 

## 4. Discussion

Our method was based on carefully curated sSNVs (ACMG recommendation-based classified pathogenic and benign). Most of the previous methods (such as DDIG, SiLVA, and TraP) were developed on limited training data (limited number of deleterious/pathogenic mutations), whereas the IDVS (identification of deleterious synonymous variants) method assumed all “likely pathogenic” sSNVs as pathogenic mutations [20]. In our development, we attempted to address these issues by using a curated dataset with an equal number of pathogenic and benign sSNVs. Such a balanced dataset seemed crucial for achieving high performance. Our selection of the RF-based model was crucial for classification. Not only did this model outperform the NB algorithm, but it also outperformed various other classification methods such as decision stump and regression models (Supplementary File 6).

The results of the feature selection can be used by other researchers in conjunction with other machine learning techniques. Thus, the analysis indicates that the splicing group features are the most indicative set. In addition, other meta-predictors such as CADD, Eigen, and TraP(V3), alongside conservation scores (PhyloP and GERP++), enhanced the pathogenicity prediction as well. Though the top 20 selected features showed the impact caused by the synonymous variant in various biological mechanisms, we were not able to identify any standout predictive patterns using these features that could uncover novel mechanisms.

The performance evaluation showed that our method outperformed all the other methods. We also demonstrated that our method was successful in classifying sSNVs associated with various disorders, such as asthma, cancer, schizophrenia, etc. The results from our classification method identified that two variants, rs2069763 and rs1130569, associated with cervical/vulvar cancer and Alzheimer’s were pathogenic, whereas the rest of the variants were classified as benign. This guided us further to use our method to reclassify all the sSNVs with clinical significance of VUS and conflicting interpretation deposited in the ClinVar database.

## 5. Conclusions

In summary, the performance of our ensemble predictor was significantly better than the other available methods in pathogenicity identification of sSNVs. In part, this was due to the use of curated training data (based on ACMG guidelines) to include only pathogenic and benign variants and to ignore other classifications. The application of our method is to provide clinical genomicists and researchers a robust technique to understand the pathogenicity and clinical relevance of a synonymous single-nucleotide variant (sSNV). Further improvement of the performance can be expected with the availability of more sSNVs clinically identified as pathogenic. This will allow a larger dataset to be used for the training of the model. 

## Figures and Tables

**Table 1:** Brief description of all the 29 features categorized into 5 groups.

Feature Class	Feature	Description
In silico predictors	CADD	It uses a c-score obtained by the integration of multiple variant annotation resources.
	EIGEN	It uses a supervised approach to derive the aggregate functional score from various annotation resources.
	TraP (V3)	It evaluates the ability of a variant to cause disease by damaging the final transcript.
Conservation Score	GERP++	GERP++ score is used to measure the conservation at the mutation position
	Phylop (100 ways)	It computes P-values for conservation-based specific lineage
	PHAST Cons	Scores based on conserved element
Codon Usage	dRSCU	Change in RSCU caused by mutation
	RSCU	RSCU (Relative synonymous codon usage) of new codon
Splicing Properties	MES	Max splice site score
	MES-KM	Has a value of 1 if site changes most or 0 if not
	dMES	Max change in splice site score
	MES-	Max splice site score decrease
	MES+	Max splice site score increase
	dpsi	The delta PSI is the predicted change in percent-inclusion due to the variant
	dpsiz	The z-score of the dPSI relative
	FAS6+	Hexamer splice suppressor motifs gained
	FAS6-	Hexamer splice suppressor motifs lost
	MEC-MC	Has a value of 1 if strongest site change or 0 if not
	MEC-CS	Has a value of 1 if a cryptic site now strongest or 0 if not
	PESS-	Octamer splice suppressor motifs lost
	PESS+	Octamer splice suppressor motifs gained
	PESE-	Octamer splice enhancer motifs lost
	PESE+	Octamer splice suppressor motifs gained
	SR-	SR-protein motifs lost
	SR+	SR-protein motifs gained
Sequence Properties	CpG_exon	Observed/expected CpG content of exon
	CpG	Has a value of 1 if mutation change a CpG or 0 if not
	f_premrna	Relative distance to end of pre-mRNA
	f_mrna	Relative distance to end of mature mRNA

**Table 2:** Statistical measures used to access the performance of classification methods. Here TP stands for true positive, FP for false positive, FN for false negative, and FP for false positive.

Statistics	Formula
Precision	$\frac{\mathrm{TP}}{\left(\mathrm{TP}\;+\;\mathrm{FP}\right)}$
Recall	$\frac{\mathrm{TP}}{\left(\mathrm{TP}\;+\;\mathrm{FN}\right)}$
F-measure	$\frac{2\;\times \;\mathrm{Precision}\;\times \;\mathrm{recall}}{\left(Precision\;+\;recall\right)}$
MCC	$\frac{\mathrm{TP}\;\times \;\mathrm{TN}\;-\;\mathrm{FN}\;\times \;\mathrm{FP}}{\sqrt{\left(\mathrm{TP}\;+\;\mathrm{FN}\right)\left(\mathrm{TP}\;+\;\mathrm{FP}\right)\left(\mathrm{TN}\;+\;\mathrm{FN}\right)\left(\mathrm{TN}\;+\;\mathrm{FP}\right)}}$
Accuracy	$\frac{\mathrm{TP}\;+\;\mathrm{TN}}{\left(\mathrm{TP}\;+\;\mathrm{FP}\;+\;\mathrm{TN}\;+\;\mathrm{FN}\right)}$
Receiver operating characteristic (ROC) curve	Plotted between TP rate to FP rate
Area under the ROC Curve (AUC)	Area Under the ROC curve, it measures the capability of a model to distinguish between classes.

The purpose of the background color is to highlight the header of the table.

**Table 3:** Summary of performance calculated using both random forest (RF) and Naive Bayes (NB) classification algorithm for 5 different training sets using 10-fold cross-validation, which includes 243 benign variants chosen randomly (5 times) along with 243 pathogenic variants. The training and testing were done using all 29 features.

	Classification Algorithm	Precision	Recall	F-Measure	MCC	Accuracy	AUC
Training Set 1	Random forest	0.886	0.802	0.842	0.703	0.849	0.929
	Naive Bayes	0.862	0.744	0.799	0.631	0.812	0.888
Training Set 2	Random forest	0.928	**0.852**	**0.888**	0.789	**0.893**	**0.959**
	Naive Bayes	0.873	0.761	0.813	0.656	0.825	0.898
Training Set 3	Random forest	**0.948**	0.831	0.886	**0.792**	**0.893**	0.941
	Naive Bayes	0.872	0.757	0.811	0.652	0.823	0.894
Training Set 4	Random forest	0.928	0.844	0.884	0.781	0.889	0.953
	Naive Bayes	0.868	0.786	0.825	0.67	0.833	0.912
Training Set 5	Random forest	0.923	0.844	0.882	0.777	0.886	0.948
	Naive Bayes	0.877	0.761	0.815	0.66	0.827	0.905

Highest value is highlighted in bold.

## Supplementary Materials

The following are available online at https://www.mdpi.com/2073-4425/11/9/1102/s1, Supplementary file 1: 5 training dataset.xlsx; Supplementary file 2: Training_dataset_Top20F.xlsx; Supplementary file 3: Sauna etal_dataset.xlsx; Supplementary file 4: Analysis_using_Trainingdataset3.docx; Supplementary file 5: Reclassification_of_VUS-CI_sSNVs.xlsx; Supplementary file 6: Supplementary file6_comparing_performing_of _more_classification_methods.docx.
