# Supplementary material for: An Ensemble Approach to Predict the Pathogenicity of Synonymous Variants
Source: Genes (Basel). 2020 Sep 21;11(9):1102. doi: 10.3390/genes11091102 (PMC7565489; doi:10.3390/genes11091102)
Supplement: Supplementary file 1 [file genes-11-01102-s001.zip › Supplementary_data/Supplementary_file_4_Analysis_using_Trainingdataset3.docx]

**Supplementary Data using training dataset 3 as training dataset**

**Table.** Summary of performance statistics calculated using random forest (RF) classification algorithm on different sets of features (top 10, top 15, top 20 ranked) using our **training dataset3** and performing 10-fold cross-validation. We observed that our selected training dataset

| **Features** | **Precision** | **Recall** | **F- Measure** | **MCC** | **Accuracy** | **AUC** |
| --- | --- | --- | --- | --- | --- | --- |
| Top 10 features | 0.906 | 0.835 | 0.869 | 0.751 | 0.874 | 0.926 |
| Top 15 features | 0.907 | 0.848 | 0.877 | 0.763 | 0.881 | 0.933 |
| Top 20 features | 0.928 | 0.844 | 0.884 | 0.781 | 0.889 | 0.940 |
| Top 20 features (Training dataset 2) | **0.933** | **0.864** | **0.897** | **0.805** | **0.912** | **0.954** |
| Top 10 features (Training dataset 2) | 0.919 | 0.844 | 0.88 | 0.772 | 0.884 | 0.938 |
| Top 15 features (Training dataset 2) | 0.912 | 0.852 | 0.881 | 0.771 | 0.885 | 0.945 |
