# Supplementary material for: An Ensemble Approach to Predict the Pathogenicity of Synonymous Variants
Source: Genes (Basel). 2020 Sep 21;11(9):1102. doi: 10.3390/genes11091102 (PMC7565489; doi:10.3390/genes11091102)
Supplement: Supplementary file 1 [file genes-11-01102-s001.zip › Supplementary_data/Supplementary file6_comparing_performing_of _more_classification_methods.docx]

**Supplementary Data comparing performing of more classification methods using all 5-training dataset.**

The comparing the performance statistics, based accuracy in distinguishing between benign and pathogenic variant along with AUC (that gives the ratio between true positive prediction rate to false positive prediction rate) value, we can infer that Random forest on training dataset performs better than other classification algorithms

|  | **Classification Algorithm** | **Precision** | **Recall** | **F- Measure** | **MCC** | **Accuracy** | **AUC** |
| --- | --- | --- | --- | --- | --- | --- | --- |
| Training set1 | Random forest | 0.886 | 0.802 | 0.842 | 0.703 | 0.849 | 0.929 |
|  | Naive Bayes | 0.862 | 0.744 | 0.799 | 0.631 | 0.812 | 0.888 |
|  | decision stump | 0.863 | 0.605 | 0.746 | 0.636 | 0.794 | 0.770 |
|  | ClassificationViaRegression | 0.870 | 0.774 | 0.819 | 0.663 | 0.829 | 0.889 |
| Training set2 | Random forest | 0.928 | **0.852** | **0.888** | 0.789 | **0.893** | **0.959** |
|  | Naive Bayes | 0.873 | 0.761 | 0.813 | 0.656 | 0.825 | 0.898 |
|  | decision stump | 0.912 | 0.617 | 0.744 | 0.613 | 0.788 | 0.784 |
|  | ClassificationViaRegression | 0.921 | 0.815 | 0.867 | 0.754 | 0.874 | 0.927 |
| Training set3 | Random forest | **0.948** | 0.831 | 0.886 | **0.792** | **0.893** | 0.941 |
|  | Naive Bayes | 0.872 | 0.757 | 0.811 | 0.652 | 0.823 | 0.894 |
|  | decision stump | 0.848 | 0.654 | 0.778 | 0.659 | 0.812 | 0.779 |
|  | ClassificationViaRegression | 0.900 | 0.819 | 0.858 | 0.731 | 0.864 | 0.920 |
| Training set4 | Random forest | 0.928 | 0.844 | 0.884 | 0.781 | 0.889 | 0.953 |
|  | Naive Bayes | 0.868 | 0.786 | 0.825 | 0.67 | 0.833 | 0.912 |
|  | decision stump | 0.825 | 0.634 | 0.753 | 0.616 | 0.792 | 0.788 |
|  | ClassificationViaRegression | 0.905 | 0.823 | 0.862 | 0.740 | 0.868 | 0.925 |
| Training set5 | Random forest | 0.923 | 0.844 | 0.882 | 0.777 | 0.886 | 0.948 |
|  | Naive Bayes | 0.877 | 0.761 | 0.815 | 0.66 | 0.827 | 0.905 |
|  | decision stump | 0.817 | 0.609 | 0.734 | 0.595 | 0.779 | 0.771 |
|  | ClassificationViaRegression | 0.916 | 0.823 | 0.873 | 0.776 | 0.876 | 0.931 |
